# Some equalities and inequalities for fusion frames

**DOI:** 10.1186/s40064-016-1685-8

**Published:** 2016-02-12

**Authors:** Qianping Guo, Jinsong Leng, Houbiao Li

**Affiliations:** School of Mathematical Sciences, University of Electronic Science and Technology of China, 611731 Chengdu, PR China

**Keywords:** Fusion frame, Equality, Frame operator

## Abstract

Fusion frames have some properties similar to those of frames in Hilbert spaces, but not all of their properties are similar. Some authors have established some equalities and inequalities for conventional frames. In this paper, we give some equalities and inequalities for fusion frames. Our results generalize and improve the remarkable results which have been obtained by Balan, Casazza and Gǎvruta etc.

## Background

Frames, which generalize the concept of bases, can take on infinitely many different representations for a given vector (Christensen 2008). Duffin and Schaeffer (1952) introduced the concept of frame to study some deep problems in nonharmonic Fourier series. After the fundamental paper by Daubechies et al. (1986), frame was popularized from then on. Now, frames are useful in some areas such as the signal and image processing, neural networks, data compression and sampling theory, among others. For signal processing frames can provide resilience to additive noise (Daubechies 1992), resilience to quantization (Goyal et al. 1998), numerical stability of reconstruction (Daubechies 1992), and greater freedom to capture signal characteristic (Benedetto and Colella 1995; Benedetto and Pfander 1998; Unser 1995).

Later on, being the generalization of the frames, fusion frames were introduced by Casazza and Kutyniok (2004) and Fornasier (2002) to handle some large systems which are impossible to handle effectively by just a simple frame. The essence of fusion frame is the construction of global frames from local frames in Hilbert space. So the characteristic fusion frame is special suiting for application such as distributing sensing, parallel processing and packet encoding, and so on. Now, many excellent results of conventional frames have been achieved and applied successfully, which properties of the conventional frames may be extended to the fusion frames? It is a tempting subject because of the complexity of the structure of fusion frames compared with conventional frames.

In this paper, we mainly study the equalities and inequalities of fusion frames. On some equalities for conventional frames were first found by Balan et al. (2007) when the authors studied the optimal decomposition of a Parseval frame. Later on, many authors such as Gǎvruţa (2006) and Zhu and Wu (2010) developed or improved some equalities or inequalities of the conventional frames on the basis of the work in originally in Balan et al. (2007).

## Preliminaries

First we will briefly recall the definitions and basic properties of fusion frames. For more details we refer to Casazza and Kutyniok (2004) and Asgari and Khosravi (2005). Throughout the paper, $\mathcal {H}$ is a Hilbert spaces, and $I=\{1,2,\ldots ,M\}$ is a subset of $\mathbf {N}$, $I_{\mathcal {H}}$ denotes the identity operator on $\mathcal {H}$.

A family of the vector $\Phi =\{\varphi _{i}\}_{i\in I}\subset \mathcal {H}$ is called a *frame*, if there exist constants $0<A\le B<\infty$ such that for any $f\in \mathcal {H}$,

1 $$\begin{aligned} A\Vert f\Vert ^{2}\le \sum \limits _{i\in I}|\langle f,\varphi _{i}\rangle |^{2}\le B\Vert f\Vert ^{2}. \end{aligned}$$

The constants *A* and *B* are known respectively as the lower and upper *frame bounds*.

### **Definition 1**

Let $\{W_{i}\}_{i\in I}$ be a sequence of closed subspaces in $\mathcal {H}$, and $\{w_{i}\}_{i\in I}$ be a family of weights, i.e., $w_{i}>0$ for all $i\in I$. Then ${\mathbf {W}}=\{(W_{i},w_{i})\}_{i\in I}$ is a *fusion frame*, if there exist constants $0<C\le D<\infty$ such that for any $f\in \mathcal {H}$

2 $$\begin{aligned} C\Vert f\Vert ^{2}\le \sum \limits _{i\in I}w_{i}\Vert \pi _{W_{i}}(f)\Vert ^{2}\le D\Vert f\Vert ^{2}, \end{aligned}$$

where $\pi _{W_{i}}$ denotes the orthogonal projection of $\mathcal {H}$ onto $W_{i}$. We call $C,\,D$ the *fusion frame bounds*. The frame ${\mathbf {W}}=\{(W_{i},w_{i})\}_{i\in I}$ is called a *tight fusion frame* if $C=D$, and is called a *Parseval fusion frame* if $C=D=1$. If we only know that ${\mathbf {W}}=\{(W_{i},w_{i})\}_{i\in I}$ satisfies the upper inequality in (2), then ${\mathbf {W}}=\{(W_{i},w_{i})\}_{i\in I}$ is called a *Bessel fusion sequence* with Bessel bound *D*.

Let $\mathbf {W}$ be a Bessel fusion sequence for $\mathcal {H}$. The *synthesis operator*$T^{*}:l^{2}(I)\rightarrow \mathcal {H}$ is defined by

$$\begin{aligned} T^{*}(\{f_{i}\}_{i\in I})=\sum \limits _{i\in I}w_{i}\pi _{W_{i}}(f_{i}),\quad \forall \{f_{i}\}_{i\in I}\in l^{2}(I). \end{aligned}$$

The adjoint operator $T:\mathcal {H}\rightarrow l^{2}(I)$ given by $T(f)=\{w_{i}\pi _{W_{i}}(f)\}_{i\in I}$ is called the *analysis operator*. In Casazza and Kutyniok (2004) we know that

$$\begin{aligned} S:\mathcal {H}\rightarrow \mathcal {H},\;Sf=\sum \limits _{i\in I}w_{i}^{2}\pi _{W_{i}}f, \end{aligned}$$

which is a bounded, self-adjoint, positive and invertible operator with $CI_{\mathcal {H}}\le S\le DI_{\mathcal {H}}$, and satisfies

$$\begin{aligned} \langle Sf,f\rangle =\sum \limits _{i\in I}w_{i}^{2}\Vert \pi _{W_{i}}f\Vert ^{2}. \end{aligned}$$

Then the following standard reconstruction formula takes places for all $f\in \mathcal {H}$,

$$\begin{aligned} f=SS^{-1}f=\sum \limits _{i\in I}w_{i}^{2}\pi _{W_{i}}(S^{-1}f), \end{aligned}$$

and

$$\begin{aligned} \langle S^{-1}f{,}\,f\rangle =\sum \limits _{i\in I}w_{i}^{2}\Vert \pi _{W_{i}}S^{-1}f\Vert ^{2}. \end{aligned}$$

Casazza and Kutyniok (2004) define the dual fusion frame of fusion frame, which is similar to the canonical dual frame in the classical frame theory.

### **Definition 2**

(*Casazza and Kutyniok*^2004^) Let $\{(W_{i},w_{i})\}_{i\in I}$ be a fusion frame with fusion frame operator *S*. Then $\{(S^{-1}W_{i},w_{i})\}_{i\in I}$ is called the *dual fusion frame* of $\{(W_{i},w_{i})\}_{i\in I}$.

If ${\mathbf {W}}=\{(W_{i},w_{i})\}_{i\in I}$ is a Bessel fusion sequence in $\mathcal {H}$, for every $J\subset I$ we define the operator $S_{J}$ by

3 $$\begin{aligned} S_{J}f=\sum \limits _{i\in J}w_{i}^{2}\pi _{W_{i}}f, \end{aligned}$$

it is trivial to show that $S_{J}$ is a self-adjoint, bounded linear operator in $\mathcal {H}$, and denote $J^{c}=I\setminus J$.

Gǎvruţa (2007) gives a more general alternate dual reconstruction formula, that is, given a fusion frame ${\mathbf {W}}=\{(W_{i},w_{i})\}_{i\in I}$ with frame operator *S* and a Bessel sequence ${\mathbf {V}}=\{(V_{i},v_{i})\}_{i\in I}$, there is

$$\begin{aligned} f=\sum \limits _{i\in I}v_{i}w_{i}\pi _{V_{i}}S^{-1}\pi _{W_{i}}f,\quad \forall f\in \mathcal {H}. \end{aligned}$$

In this case we call ${\mathbf {V}}=\{(V_{i},v_{i})\}_{i\in I}$ an *alternate dual fusion frame* of ${\mathbf {W}}=\{(W_{i},w_{i})\}_{i\in I}$.

In the study of longstanding conjecture of signal processing community: a signal can be reconstructed without information about the phase. Balan et al. (2006) found some new frame equalities. In order to compare with the “Main results” section, we list the important equalities in Balan et al. (2007) as follows.

### **Theorem 1**

(Balan et al. 2007)* Let*$\{f_{i}\}_{i\in I}$* be a Parseval frame for*$\mathcal {H}$.* Then for any*$J\subset I$* and*$f\in \mathcal {H}$* we have*

4 $$\begin{aligned}&\sum \limits _{i\in J}|\langle f,f_{i}\rangle |^{2}-\Vert \sum \limits _{i\in J}\langle f,f_{i}\rangle f_{i}\Vert ^{2}\nonumber \\&=\sum \limits _{i\in J^{c}}|\langle f,f_{i}\rangle |^{2}-\Vert \sum \limits _{i\in J^{c}}\langle f,f_{i}\rangle f_{i}\Vert ^{2}. \end{aligned}$$

### *Remark 1*

A frame $\{g_{i}\}_{i\in I}$ is called alternate dual frame of $\{f_{i}\}_{i\in I}$ and $f=\sum \limits _{i\in I}\langle f,g_{i}\rangle f_{i}$, $f\in \mathcal {H}$. Then we get a more general result about the alternate dual frame (Gǎvruţa 2006).

### **Theorem 2**

*Let*$\{f_{i}\}_{i\in I}$* be a frame for*$\mathcal {H}$* with an alternate dual frame*$\{g_{i}\}_{i\in I}\subset \mathcal {H}$.* Then for any*$J\subset I$* and any*$f\in \mathcal {H}$* we have*

5 $$\begin{aligned} \begin{array}{l} Re\left( \sum \limits _{i\in J}\langle f,g_{i}\rangle \overline{\langle f,f_{i}\rangle }\right) - \Vert \sum \limits _{i\in J}\langle f,g_{i}\rangle f_{i}\Vert ^{2}\\ =Re\left( \sum \limits _{i\in J^{c}}\langle f,g_{i}\rangle \overline{\langle f,f_{i}\rangle }\right) -\Vert \sum \limits _{i\in J^{c}}\langle f,g_{i}\rangle f_{i}\Vert ^{2}. \end{array} \end{aligned}$$

Zhu and Wu (2010) generalized the equality (5) to a more general form which does not involve the real parts of the complex numbers.

### **Theorem 3**

*Let*$\{f_{i}\}_{i\in I}$* be a frame for*$\mathcal {H}$* and*$\{g_{i}\}_{i\in I}\subset \mathcal {H}$* is an alternate dual frame of*$\{f_{i}\}_{i\in I}$.* Then for any*$J\subset I$* and*$f\in \mathcal {H}$* we have*

6 $$\begin{aligned} \begin{array}{l} \left( \sum \limits _{i\in J}\langle f,g_{i}\rangle \overline{\langle f,f_{i}\rangle }\right) -\Vert \sum \limits _{i\in J}\langle f,g_{i}\rangle f_{i}\Vert ^{2}\\ =\overline{\left( \sum \limits _{i\in J^{c}}\langle f,g_{i}\rangle \overline{\langle f,f_{i}\rangle }\right) } -\Vert \sum \limits _{i\in J^{c}}\langle f,g_{i}\rangle f_{i}\Vert ^{2}. \end{array} \end{aligned}$$

Next, we extended this equality to fusion frame.

## Main results

Motivated by the work of Balan et al. (2007) and Gǎvruţa (2006), in this section, we continue this work about fusion frames and get some important equalities and inequalities of these frames in a different case.

### **Lemma 1**

(Zhu and Wu 2010)* LetP andQ be two linear bounded operators on*$\mathcal {H}$* such that*$P+Q=I_{\mathcal {H}}$.* Then*$P-P^{*}P=Q^{*}-Q^{*}Q$.

Now, we present main theorems of this section.

### **Theorem 4**

*Let*$\{(W_{i},w_{i})\}_{i\in I}$* be a fusion frame for*$\mathcal {H}$* with the fusion frame operatorS*, $\{(V_{i},v_{i})\}_{i\in I}$* is the alternate dual fusion frame of*$\{(W_{i},w_{i})\}_{i\in I}$.* Then, for any*$J\subset I$* and any*$f\in \mathcal {H}$,

$$\begin{aligned} \begin{array}{lll} \sum \limits _{i\in J}v_{i}w_{i}\langle S^{-1}\pi _{W_{i}}(f),\pi _{V_{i}}f\rangle -\Vert \sum \limits _{i\in J}v_{i}w_{i}\pi _{V_{i}}S^{-1}\pi _{W_{i}}f\Vert ^{2}&{}\\ = \sum \limits _{i\in J^{c}}v_{i}w_{i}\langle \pi _{V_{i}}f,S^{-1}\pi _{W_{i}}f\rangle -\Vert \sum \limits _{i\in J^{c}}v_{i}w_{i}\pi _{V_{i}}S^{-1}\pi _{W_{i}}f\Vert ^{2}. \end{array} \end{aligned}$$

### *Proof*

For any $J\subset I$, we define a bounded linear operator $S_{J}$ as

$$\begin{aligned} S_{J}f=\sum \limits _{i\in J}v_{i}w_{i}\pi _{V_{i}}S^{-1}\pi _{W_{i}}f,\quad \forall f\in \mathcal {H}. \end{aligned}$$

Clearly, $S_{J}+S_{J^{c}}=I_{\mathcal {H}}$. This, together with Lemma 1, implies that

7 $$\begin{aligned} \begin{array}{l} \sum \limits _{i\in J}v_{i}w_{i}\langle S^{-1}\pi _{W_{i}}(f),\pi _{V_{i}}f\rangle -\Vert \sum \limits _{i\in J}v_{i}w_{i}\pi _{V_{i}}S^{-1}\pi _{W_{i}}f\Vert ^{2}\\ =\sum \limits _{i\in J}v_{i}w_{i}\langle S^{-1}\pi _{W_{i}}(f),\pi _{V_{i}}f\rangle -\langle S_{J}f,S_{J}f\rangle \\ =\langle S_{J}f,f\rangle -\langle S_{J}^{*}S_{J}f,f\rangle \\ =\langle S_{J^{c}}^{*}f,f\rangle -\langle S_{J^{c}}^{*}S_{J^{c}}f,f\rangle \\ =\langle f,S_{J^{c}}f\rangle -\langle S_{J^{c}}f,S_{J^{c}}f\rangle \\ =\langle f,\sum \limits _{i\in J^{c}}v_{i}w_{i}\pi _{V_{i}}S^{-1}\pi _{W_{i}}f\rangle \\ \quad -\,\Vert \sum \limits _{i\in J^{c}}v_{i}w_{i}\pi _{V_{i}}S^{-1}\pi _{W_{i}}f\Vert ^{2}\\ =\sum \limits _{i\in J^{c}}v_{i}w_{i}\langle \pi _{V_{i}}f,S^{-1}\pi _{W_{i}}f\rangle \\ \quad -\,\Vert \sum \limits _{i\in J^{c}}v_{i}w_{i}\pi _{V_{i}}S^{-1}\pi _{W_{i}}f\Vert ^{2}. \end{array} \end{aligned}$$

In the situation of Parseval fusion frames the equality is of special form.□

### **Corollary 1**

*Let*$\{(W_{i},w_{i})\}_{i\in I}$* be a Parseval fusion frame for*$\mathcal {H}$* with the fusion frame operator*$S=I_{\mathcal {H}}$, $\{(V_{i},v_{i})\}_{i\in I}$* is the alternate dual fusion frame of*$\{(W_{i},w_{i})\}_{i\in I}$.* Then, for any*$J\subset I$* and any*$f\in \mathcal {H}$,

$$\begin{aligned} \begin{array}{lll} \sum \limits _{i\in J}v_{i}w_{i}\langle \pi _{W_{i}}(f),\pi _{V_{i}}f\rangle -\Vert \sum \limits _{i\in J}v_{i}w_{i}\pi _{V_{i}}\pi _{W_{i}}f\Vert ^{2}&{}\\ \quad = \sum \limits _{i\in J^{c}}v_{i}w_{i}\langle \pi _{V_{i}}f,\pi _{W_{i}}f\rangle -\Vert \sum \limits _{i\in J^{c}}v_{i}w_{i}\pi _{V_{i}}\pi _{W_{i}}f\Vert ^{2}. \end{array} \end{aligned}$$

### *Remark 2*

Clearly, when the dual fusion frame of $\{(W_{i},w_{i})\}_{i\in I}$ is itself, i.e., $\{(V_{i},v_{i})\}_{i\in I}=\{(W_{i},w_{i})\}_{i\in I}$, which was obtained Theorem 2.2 in Xiyan et al. (2009) as a particular case from the above result.

In fact, similarly to the proof of Theorem 4, we can give a more general result as follow. Moreover, the result has another proof in Xiao et al. (2014).

### **Theorem 5**

*Let*$\{(W_{i},w_{i})\}_{i\in I}$* be a fusion frame for*$\mathcal {H}$* with the fusion frame operatorS*, $\{(V_{i},v_{i})\}_{i\in I}$* is the alternate dual fusion frame of*$\{(W_{i},w_{i})\}_{i\in I}$.* Then, for any*$f\in \mathcal {H}$* and any*$\{b_{i}\}_{i\in I}\in l^{\infty }(I)$,

8 $$\begin{aligned} \begin{array}{l} \sum \limits _{i\in I}b_{i}v_{i}w_{i}\langle S^{-1}\pi _{W_{i}}f,\pi _{V_{i}}f\rangle \\ \quad -\,\Vert \sum \limits _{i\in I}b_{i}v_{i}w_{i}\pi _{V_{i}}S^{-1}\pi _{W_{i}}f\Vert ^{2}\\ =\sum \limits _{i\in I}(1-\bar{b_{i}})v_{i}w_{i}\langle \pi _{V_{i}}f,S^{-1}\pi _{W_{i}}f\rangle \\ \quad -\,\Vert \sum \limits _{i\in I}(1-b_{i})v_{i}w_{i}\pi _{V_{i}}S^{-1}\pi _{W_{i}}f\Vert ^{2}. \end{array} \end{aligned}$$

where $\bar{b_{i}}$ is the conjugata of $b_{i}$.

### *Remark 3*

Let $\{(W_{i},w_{i})\}_{i\in I}$ be a tight fusion frame for $\mathcal {H}$ with the fusion frame bound *A*, and $b_{i}$ is real for any $i\in I$. In this case, using the Theorem 5, we obtain

$$\begin{aligned} \begin{array}{l} A\sum \limits _{i\in I}b_{i}v_{i}w_{i}\langle S^{-1}\pi _{W_{i}}f,\pi _{V_{i}}f\rangle \\ \quad -\,\Vert \sum \limits _{i\in I}b_{i}v_{i}w_{i}\pi _{V_{i}}S^{-1}\pi _{W_{i}}f\Vert ^{2}\\ =A\sum \limits _{i\in I}(1-b_{i})v_{i}w_{i}\langle \pi _{V_{i}}f,S^{-1}\pi _{W_{i}}f\rangle \\ \quad -\,\Vert \sum \limits _{i\in I}(1-b_{i})v_{i}w_{i}\pi _{V_{i}}S^{-1}\pi _{W_{i}}f\Vert ^{2}. \end{array} \end{aligned}$$

### **Lemma 2**

(Gǎvruţa 2006)* LetP andQ are two self-adjoint bounded linear operators in*$\mathcal {H}$* and*$P+Q=I_{\mathcal {H}}$.* Then we have*

$$\begin{aligned} \langle Pf,f\rangle +\Vert Qf\Vert ^{2}=\langle Qf,f\rangle +\Vert Pf\Vert ^{2}\ge \frac{3}{4}\langle f,f\rangle . \end{aligned}$$

### **Theorem 6**

*Let*$\{(W_{i},w_{i})\}_{i\in I}$* be a fusion frame for*$\mathcal {H}$* with the fusion frame operatorS*, $\{(S^{-1}W_{i},w_{i})\}_{i\in I}$* is the dual fusion frame of*$\{(W_{i},w_{i})\}_{i\in I}$.* Then, for any*$J\subset I$* and any*$f\in \mathcal {H}$,* we have*

$$\begin{aligned} \begin{array}{l} \sum \limits _{i\in J}w_{i}^{2}\Vert \pi _{W_{i}}f\Vert ^{2}+\sum \limits _{i\in I}w_{i}^{2}\Vert \pi _{W_{i}}S^{-1}S_{J^{c}}f\Vert ^{2}\\ =\sum \limits _{i\in J^{c}}w_{i}^{2}\Vert \pi _{W_{i}}f\Vert ^{2}+\sum \limits _{i\in I}w_{i}^{2}\Vert \pi _{W_{i}}S^{-1}S_{J}f\Vert ^{2}\\ \ge \displaystyle \frac{3}{4}\langle Sf,f\rangle . \end{array} \end{aligned}$$

### *Proof*

Applying $S=S_{J}+S_{J^{c}}$, we have that $I_{\mathcal {H}}=S^{-\frac{1}{2}}S_{J}S^{-\frac{1}{2}}+S^{-\frac{1}{2}}S_{J^{c}}S^{-\frac{1}{2}}$. Combining this with Lemma 2, it follows that

9 $$\begin{aligned} \begin{array}{l} \langle S^{-\frac{1}{2}}S_{J}S^{-\frac{1}{2}}f,f\rangle +\Vert S^{-\frac{1}{2}}S_{J^{c}}S^{-\frac{1}{2}}f\Vert ^{2} \\=\langle S^{-\frac{1}{2}}S_{J^{c}}S^{-\frac{1}{2}}f,f\rangle +\Vert S^{-\frac{1}{2}}S_{J}S^{-\frac{1}{2}}f\Vert \\\ge \frac{3}{4}\langle f,f\rangle . \end{array} \end{aligned}$$

Replacing *f* by $S^{\frac{1}{2}}f$, one has

$$\begin{aligned} \begin{array}{l} \langle S_{J}f,f\rangle +\langle S^{-1}S_{J^{c}}f,S_{J^{c}}f\rangle \\=\langle S_{J^{c}}f,f\rangle +\langle S^{-1}S_{J}f,S_{J}f\rangle \ge \frac{3}{4}\langle Sf,f\rangle . \end{array} \end{aligned}$$

Combining this with $\langle S_{J}f,f\rangle =\sum \limits _{i\in J}w_{i}^{2}\Vert \pi _{w_{i}}f\Vert ^{2}$ and $\langle S^{-1}f,f\rangle =\sum \limits _{i\in I}w_{i}^{2}\Vert \pi _{W_{i}}S^{-1}f\Vert ^{2}$, the proof is completed.□

### *Remark 4*

The identity of above was established Theorem 2.1 in Xiyan et al. (2009), but the inequality in this form is a new result.

### **Corollary 2**

*Let*$\{(W_{i},w_{i})\}_{i\in I}$* be a tight fusion frame for*$\mathcal {H}$* with the fusion frame boundA*.* Then*

$$\begin{aligned} \begin{array}{l} A\sum \limits _{i\in J}w_{i}^{2}\Vert \pi _{W_{i}}f\Vert ^{2}+\Vert S_{J^{c}}f\Vert ^{2} \\=A\sum \limits _{i\in J^{c}}w_{i}^{2}\Vert \pi _{W_{i}}(f)\Vert ^{2}+\Vert S_{J}f\Vert ^{2}\\ \ge \frac{3}{4}A^{2}\langle f,f\rangle . \end{array} \end{aligned}$$

*In addition, if*$\{(W_{i},w_{i})\}_{i\in I}$* is a Parseval fusion frame for*$\mathcal {H}$*, then we have*

10 $$\begin{aligned} \begin{array}{l} \sum \limits _{i\in I}w_{i}^{2}\Vert \pi _{W_{i}}f\Vert ^{2}+\Vert S_{J^{c}}f\Vert ^{2}\\ =\sum \limits _{i\in J^{c}}w_{i}^{2}\Vert \pi _{W_{i}}(f)\Vert ^{2}+\Vert S_{J}f\Vert ^{2}\\ \ge \frac{3}{4}\langle f,f\rangle . \end{array} \end{aligned}$$

### *Proof*

Since $\{(W_{i},w_{i})\}_{i\in I}$ be a tight fusion frame for $\mathcal {H}$ with the fusion frame bound *A*, then for any $f\in \mathcal {H}$,

$$\begin{aligned} \sum \limits _{i\in J}w_{i}^{2}\Vert \pi _{W_{i}}f\Vert ^{2}=\langle Sf,f\rangle =A\Vert f\Vert ^{2},\,\pi _{W_{i}}S^{-1}=\frac{1}{A}\pi _{W_{i}}, \end{aligned}$$

and

$$\begin{aligned} \sum \limits _{i\in I}w_{i}^{2}\Vert \pi _{W_{i}}S^{-1}S_{J}f\Vert ^{2}=\frac{1}{A}\Vert S_{J}f\Vert ^{2}. \end{aligned}$$

It follows from Theorem 6 that, for any $f\in \mathcal {H}$,

$$\begin{aligned} \begin{array}{l} A\sum \limits _{i\in J}w_{i}^{2}\Vert \pi _{W_{i}}f\Vert ^{2}+\Vert S_{J^{c}}f\Vert ^{2} \\=A\sum \limits _{i\in J^{c}}w_{i}^{2}\Vert \pi _{W_{i}}(f)\Vert ^{2}+\Vert S_{J}f\Vert ^{2} \\\ge \frac{3}{4}A^{2}\langle f, f\rangle . \end{array} \end{aligned}$$

□

### **Theorem 7**

*Let*$\{(W_{i},w_{i})\}_{i\in I}$* be a tight fusion frame for*$\mathcal {H}$* with the fusion frame boundA*.* Then, for any*$J,\,E\subset I$* with*$J\cap E=\emptyset$,* and any*$f\in \mathcal {H}$,* we have*

11 $$\begin{aligned} \begin{array}{l} \Vert S_{J\cup E}f\Vert ^{2}-\Vert S_{J^{c}\setminus E}f\Vert ^{2} \\=\Vert S_{J}f\Vert ^{2}-\Vert S_{J^{c}}f\Vert ^{2}+ 2A\sum \limits _{i\in E}w_{i}^{2}\Vert \pi _{W_{i}}f\Vert ^{2}. \end{array} \end{aligned}$$

### *Proof*

Applying Corollary 2 yields that

$$\begin{aligned} \begin{array}{l} \Vert S_{J\cup E}f\Vert ^{2}-\Vert S_{J^{c}\setminus E}f\Vert ^{2}\\ \quad =A\sum \limits _{i\in J\cup E}w_{i}^{2}\Vert \pi _{W_{i}}f\Vert ^{2}- A\sum \limits _{i\in J^{c}\setminus E}w_{i}^{2}\Vert \pi _{W_{i}}f\Vert ^{2}\\ \quad =A\sum \limits _{i\in J}w_{i}^{2}\Vert \pi _{W_{i}}f\Vert ^{2}- A\sum \limits _{i\in J^{c}}w_{i}^{2}\Vert \pi _{W_{i}}f\Vert ^{2}\\ \qquad +\,2A\sum \limits _{i\in E}w_{i}^{2}\Vert \pi _{W_{i}}f\Vert ^{2}\\ \quad =\Vert S_{J}f\Vert ^{2}-\Vert S_{J^{c}}f\Vert ^{2}+2A\sum \limits _{i\in E}w_{i}^{2}\Vert \pi _{W_{i}}f\Vert ^{2}. \end{array} \end{aligned}$$

Similarly Corollary 3.6 in Xiao and Zeng (2010), obtain

### **Corollary 3**

*Let*$\{(W_{i},w_{i})\}_{i\in I}$* be a tight fusion frame for*$\mathcal {H}$* with the fusion frame boundA*.* Then, for any*$J_{i}\subset I,\,(i\in N)$,* where*$N\ge 2$* is a positive integer, with*$J_{i}\cap J_{j}=\emptyset$,* for*$i\ne j$, $I=\cup _{i=1}^{N}J_{i}$.* Then for any*$f\in \mathcal {H}$, *we have*

$$\begin{aligned} \begin{array}{l} \Vert S_{(\cup _{i=N_{1}}^{N_{4}}J_{i})}f\Vert ^{2}-\Vert S_{(\cup _{i=1}^{N_{1}-1}J_{i}+\cup _{i=N_{4}-1}^{N}J_{i})}f\Vert ^{2}\\ =\Vert S_{(\cup _{i=N_{2}}^{N_{3}}J_{i})}f\Vert ^{2}-\Vert S_{(\cup _{i=N_{2}}^{N_{3}}J_{i})^{c}}f\Vert ^{2}\\ +\,2A\sum _{(\cup _{i=N_{1}}^{N_{2}-1}+\cup _{i=N_{3}+1}^{N_{4}})}w_{i}^{2}\Vert \pi _{W_{i}}f\Vert ^{2}, \end{array} \end{aligned}$$

where $N_{i},\,(1\le i\le 4)$ are positive integers satisfying $1\le N_{1}\le N_{2}<N_{3}<N_{4}\le N-1$.

### *Proof*

Applying (11), replace *J* and *E* by $\cup _{i=N_{2}}^{N_{3}}J_{i}$ and $\cup _{i=N_{1}}^{N_{2}-1}J_{i}+\cup _{i=N_{3}+1}^{N_{4}}J_{i}$, the above result hold.□

The inequality (10) in Corollary 2 leads us to introduce some notations $v_{-}(\mathbf {W},J)$ and $v_{+}(\mathbf {W},J)$. Let ${\mathbf {W}}=\{(W_{i},w_{i})\}_{i\in I}$ be a Parseval fusion frame. For any $J\subset I$ and $f\in \mathcal {H}$, define

$$\begin{aligned} v_{+}({\mathbf {W}},J)=\sup _{f\ne 0}\frac{\sum \limits _{i\in J^{c}}w_{i}^{2}\Vert \pi _{W_{i}}f\Vert ^{2}+\Vert \sum \limits _{i\in J}w_{i}^{2}\pi _{w_{i}}f\Vert ^{2}}{\Vert f\Vert ^{2}}, \end{aligned}$$

and

$$\begin{aligned} v_{-}({\mathbf {W}},J)=\inf _{f\ne 0}\frac{\sum \limits _{i\in J^{c}}w_{i}^{2}\Vert \pi _{W_{i}}f\Vert ^{2}+\Vert \sum \limits _{i\in J}w_{i}^{2}\pi _{w_{i}}f\Vert ^{2}}{\Vert f\Vert ^{2}}. \end{aligned}$$

### **Theorem 8**

$v_{-}({\mathbf {W}},J)$* and*$v_{+}({\mathbf {W}},J)$* have the following properties:*

1. $$\frac{3}{4}\le v_{-}({\mathbf {W}},J)\le v_{+}({\mathbf {W}},J)\le 1$$;
2. $$v_{-}({\mathbf {W}},J^{c})=v_{-}({\mathbf {W}},J)$$, $$v_{+} ({\mathbf {W}},J^{c})=v_{+}({\mathbf {W}},J)$$;
3. $$v_{-}({\mathbf {W}},J)=v_{+}({\mathbf {W}},J)$$, $$v_{-}({\mathbf {W}},\emptyset )=v_{+}({\mathbf {W}},\emptyset )$$.

### *Proof*

By inequality (10), $\frac{3}{4}\le v_{-}({\mathbf {W}},J)$ holds trivially.

For any $f, g\in \mathcal {H}$ and any $J\subset I$, we have

$$\begin{aligned} \begin{array}{lll} \Vert \sum \limits _{i\in J}w_{i}^{2}\pi _{w_{i}}f\Vert ^{2}&{}=&{} \sup \limits _{\Vert g\Vert =1}|\langle \sum \limits _{i\in J}w_{i}^{2}\pi _{w_{i}}f,g\rangle |^{2}\\ &{}=&{}\sup \limits _{\Vert g\Vert =1}|\sum \limits _{i\in J}w_{i}^{2}\langle \pi _{w_{i}}f,\pi _{w_{i}}g\rangle |^{2}\\ &{}\le &{}\sup \limits _{\Vert g\Vert =1}\sum \limits _{i\in J}w_{i}^{2} \Vert \pi _{w_{i}}f\Vert ^{2}\sum \limits _{i\in J}w_{i}^{2}\Vert \pi _{w_{i}}g\Vert ^{2}\\ &{}=&{}\sup \limits _{\Vert g\Vert =1}\Vert g\Vert ^{2}\sum \limits _{i\in J}w_{i}^{2}\Vert \pi _{w_{i}}f\Vert ^{2}\\ &{}=&{}\sum \limits _{i\in J}w_{i}^{2}\Vert \pi _{w_{i}}f\Vert ^{2}. \end{array} \end{aligned}$$

Hence,

$$\begin{aligned} \begin{array}{l} \sum \limits _{i\in J^{c}}w_{i}^{2}\Vert \pi _{W_{i}}f\Vert ^{2}+ \Vert \sum \limits _{i\in J}w_{i}^{2}\pi _{w_{i}}f\Vert ^{2}\\ \le \sum \limits _{i\in J^{c}}w_{i}^{2}\Vert \pi _{W_{i}}f\Vert ^{2}+ \sum \limits _{i\in J}w_{i}^{2}\Vert \pi _{w_{i}}f\Vert ^{2}\le \Vert f\Vert ^{2}, \end{array} \end{aligned}$$

This implies that $\Vert \sum \limits _{i\in J}w_{i}^{2}\pi _{w_{i}}f\Vert ^{2}\le \sum \limits _{i\in J}w_{i}^{2}\Vert \pi _{w_{i}}f\Vert ^{2}$. That is $v_{+}({\mathbf {W}},J)\le 1$.

(2) and (3) follow directly by inequality (10) in Corollary 2.□

Some results for the Parseval fusion frame were established in Xiyan et al. (2009). For the reader’s convenience and our results equivalence, we not only recall its formulation but also provide its proof as follows.

### **Theorem 9**

*Let*${\mathbf {W}}=\{(W_{i},w_{i})\}_{i\in I}$* be a Parseval fusion frame for*$\mathcal {H}$.* Then, for any*$J\subset I$* and any*$f\in \mathcal {H}$,* the following statements are equivalent:*

1. $$v_{-}({\mathbf {W}},J)=v_{+}({\mathbf {W}},J)=1$$;
2. $$\sum \limits _{i\in J}w_{i}^{2}\Vert \pi _{w_{i}}f\Vert ^{2}= \Vert \sum \limits _{i\in J}w_{i}^{2}\pi _{w_{i}}f\Vert ^{2}$$;
3. $$\sum \limits _{i\in J^{c}}w_{i}^{2}\Vert \pi _{w_{i}}f\Vert ^{2}= \Vert \sum \limits _{i\in J^{c}}w_{i}^{2}\pi _{w_{i}}f\Vert ^{2}$$;
4. $$S_{J}S_{J^{c}}f=0$$.

### *Proof*

(1)$\Rightarrow$ (2). Since $\mathbf {W}$ is a Parseval fusion frame, then for any $f\in \mathcal {H}$, we have $\sum \limits _{i\in J^{c}}w_{i}^{2}\Vert \pi _{W_{i}}f\Vert ^{2}+\sum \limits _{i\in J}w_{i}^{2} \Vert \pi _{w_{i}}f\Vert ^{2}=\Vert f\Vert ^{2}$. This implies that

$$\begin{aligned} \begin{array}{lll} \sum \limits _{i\in J}w_{i}^{2}\Vert \pi _{w_{i}}f\Vert ^{2}&{}=&{}\Vert f\Vert ^{2}- \sum \limits _{i\in J^{c}}w_{i}^{2}\Vert \pi _{W_{i}}f\Vert ^{2}\\ &{}=&{}\sum \limits _{i\in J^{c}}w_{i}^{2}\Vert \pi _{W_{i}}f\Vert ^{2}+ \Vert \sum \limits _{i\in J}w_{i}^{2}\pi _{w_{i}}f\Vert ^{2}\\ &{}&{}-\sum \limits _{i\in J^{c}}w_{i}^{2}\Vert \pi _{W_{i}}f\Vert ^{2}\\ &{}=&{}\Vert \sum \limits _{i\in J}w_{i}^{2}\pi _{w_{i}}f\Vert ^{2}. \end{array} \end{aligned}$$

Applying (10), (3) $\Leftarrow$ (2) $\Rightarrow$ (1) hold trivially.

(2)$$\Leftrightarrow$$ (4) follows from

$$\begin{aligned} \begin{array}{lll} \sum \limits _{i\in J}w_{i}^{2}\Vert \pi _{w_{i}}f\Vert ^{2}-\Vert \sum \limits _{i\in J}w_{i}^{2} \pi _{w_{i}}f\Vert ^{2}\\ =\langle S_{J}f,f\rangle -\langle S_{J}f,S_{J}f\rangle =\langle (S_{J}-S_{J}^{2})f,f\rangle \\ =\langle S_{J}(I-S_{J})f,f\rangle =\langle S_{J}S_{J^{c}}f,f\rangle . \end{array} \end{aligned}$$

□

## Conclusions

In frame theory, fusion frames have some properties similar to those of frames in Hilbert spaces, but not all of their properties are similar. Many excellent results of frames have been achieved and applied successfully, which properties of the frames may be extended to the fusion frames, which requires a lot of efforts to deal with. In this paper, we extend some equalities and inequalities of the frame to the fusion frames, which generalize and improve the remarkable results which have been obtained.
